# 4-Hexylresorcinol Treatment before Degumming Increases the β-Sheet Structure of Silk Sericin and BMP-2 Expression in RAW264.7 Cells

**DOI:** 10.3390/ijms24010150

**Published:** 2022-12-21

**Authors:** Ji Hae Lee, HaeYong Kweon, Ji-Hyeon Oh, Yei-Jin Kang, Dae-Won Kim, Won-Geun Yang, Weon-Sik Chae, Seong-Gon Kim

**Affiliations:** 1 Industrial Insect and Sericulture Division, National Institute of Agricultural Sciences, RDA, Wanju 55365, Republic of Korea; 2 Department of Oral and Maxillofacial Surgery, College of Dentistry, Gangneung-Wonju National University, Gangneung 28644, Republic of Korea; 3 Department of Oral Biochemistry, College of Dentistry, Gangneung-Wonju National University, Gangneung 28644, Republic of Korea; 4 Daegu Center, Korea Basic Science Institute, Daegu 41566, Republic of Korea

**Keywords:** sericin, 4-hexylresorcinol, bone graft, BMP-2

## Abstract

Silk sericin is a degumming product used by the silk industry. The degumming process can affect the protein structure and molecular weight of silk sericin. The present study examined how pretreatment with 4-hexylresorcinol (4HR) affects the biomedical properties of silk sericin. Before the degumming process, silkworm cocoons were treated with 4HR solution. The protein structure of the final degumming product was evaluated by Fourier transform infrared spectroscopy (FT-IR) and scanning electron microscopy. Untreated silk sericin (S) and silk sericin pretreated with 4HR (S+4HR) were added to RAW264.7 cells, and the expression of BMP-2 was determined. The bone-regenerating capacity of S+4HR was evaluated using the critical-sized rat calvarial defect model. Compared with S, S+4HR showed an increase in β-sheet structures. Administration of S+4HR to RAW264.7 cells increased expression of BMP-2, mainly via the TLR-mediated signaling pathway. Bone volume, as measured by micro-computerized tomography, was significantly greater in the S+4HR group than in the S, gelatin alone, and unfilled control groups (*p* < 0.05 each). Expression of BMP-2 and runx2 in tissue specimens was significantly higher following treatment with S+4HR than with S (*p* < 0.05). Taken together, these findings show that 4HR pretreatment before the degumming process increased the β-sheet structure of silk sericin, as well as inducing BMP-2 expression and bone regeneration ability.

## 1. Introduction

Silk mats, which are composed of silk sericin and silk fibroin, have shown success in guided bone regeneration, both in animals [1] and in clinical trials [2]. Silk sericin is the main component of silk mats that is responsible for bone regeneration [1,3]. In addition to its use in bone grafts, silk sericin has been used in wound dressings, cosmetics, and food [4]. The form of silk sericin should depend on its intended use. For example, when used as a food supplement, silk sericin will be digested in the gastrointestinal tract and absorbed as single amino acids or peptides. Thus, the conformation and molecular weight of silk sericin in the final commercial product may not be an important consideration in its production. When used in bone grafting, however, silk sericin will interact directly with cells, making the sericin extraction process important when manufacturing silk sericin for grafting.

The process by which sericin is extracted from silkworm cocoons is called degumming [5]. Many types of degumming processes have been used to remove silk sericin from silk fibroin, with these processes differing depending on the intended application of silk sericin. As a biomedical resource, silk sericin can be released from silk mats in fragmented form, which can be used for bone regeneration [1]. Moreover, sericin used in biomedical applications should be extracted by a biologically inert process.

A new degumming process to produce silk sericin for use in bone grafts involves extraction at low temperature (37 °C–60 °C), along with sonification using a centrifuge [5]. Silk sericin extracted using this technique has a structure comprising abundant β-sheets with high molecular weight (M.W. > 30 kDa) [5]. This form of sericin can induce expression of bone morphogenic protein-2 (BMP-2) in RAW264.7 cells via a Toll-like receptor (TLR) mediated signaling pathway [5,6]. Gelatin sponges containing this type of sericin show a higher level of bone regeneration than untreated control sponges [5]. One disadvantage of this extraction technique, however, is its low yield, with the weight of biologically active sericin being <1% of the initial weight of silkworm cocoons (unpublished data). In addition, the sonification time is relatively long at 2 h, and the cellulose membrane used for separation is expensive.

4-Hexylresorcinol (4HR) is a resorcinolic lipid that is used in antiseptics and as a food additive [7]. When used in cosmetics, the combination of 4HR plus niacinamide results in better skin tone and anti-aging effects than niacinamide alone [8]. The biological activity of 4HR is broad. In addition to being a Class I and Class II histone deacetylase inhibitor and a tyrosinase inhibitor [7], 4HR can inhibit the NF-κB pathway and the production of inflammatory cytokines [9]. 4HR was recently shown to bind to mutant p53 protein and alter its structure from a random coil to a β-sheet [10]. 4HR treatment of mutant p53 protein can rescue its DNA binding ability and p53 transcription activity [10]. In addition, 4HR pretreatment enhances the structural stability of DNA [11].

Boiling alters protein conformation. The classical degumming process includes a boiling step, suggesting that this process can also alter protein conformation. If 4HR pretreatment enhances the conformational stability of silk sericin, then silk sericin extracted from 4HR-pretreated silkworm cocoons would show increased β-sheet formation and an ability to induce BMP-2 similar to that of silk sericin extracted at low temperatures.

The purpose of this study was to evaluate the effect of 4HR pretreatment before the degumming process on silk sericin. Successful incorporation of 4HR into sericin was evaluated by FT-IR and by analysis of protein release. The biological effects of 4HR-pretreated sericin were analyzed using a murine macrophage cell line and an animal model.

## 2. Results

### 2.1. 4HR Pretreatment Increases β-Sheet Conformation in Silk Sericin Despite Degumming at High Temperature

**Figure 1:**
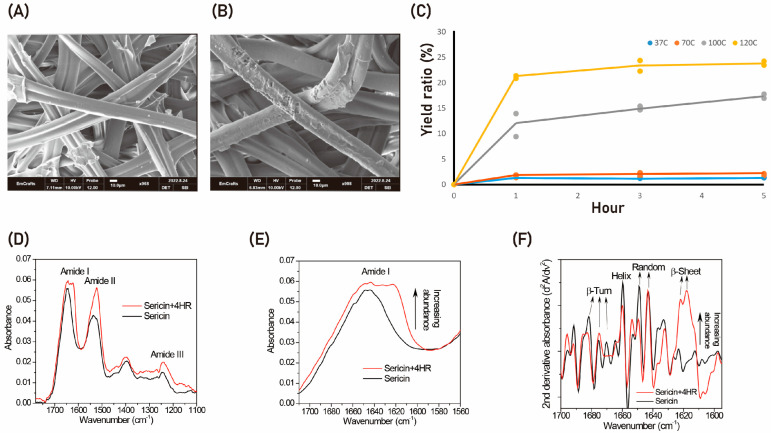
The effect of 4HR pretreatment before degumming. (**A**) Micro-computerized tomography (mCT) of an untreated cocoon, showing silk fibroin covered by silk sericin. (**B**) mCT of a cocoon treated with 0.5% 4HR in ethanol, showing partial damage to the silk sericin coating, although most of the pretreated cocoon looked similar to the untreated cocoon. (**C**) Dependence of the yield ratio of collected sericin after degumming on the temperature of the degumming solution and the duration of degumming. (**D**–**F**) FT-IR analysis, showing (**D**) that the amide I band, which corresponds to C=O stretching, was highly dependent on the conformation of protein secondary structures; (**E**) that 4HR pretreatment of silk sericin resulted in increased absorption at ~1620 cm^−1^; and (**F**) that the broad amide I band could be split into several fine peaks by calculating the second derivative of the infrared absorption spectrum. In addition, the abundance of β-sheet secondary structure at ~1620 cm^−1^ was higher, whereas the abundances of random, helix, and β-turn structures (at 1649, 1660, and 1682 cm^−1^, respectively) were markedly lower, in 4HR treated than in untreated sericin protein.

SEM showed that 4HR pretreatment had no significant effect on silkworm cocoons (Figure 1A,B). After three ethanol washes, the concentration of 4HR incorporated into pretreated cocoons was 9.7 ± 1.8 mg/g. The yield ratio after the degumming process was increased by increasing the temperature of the process (Figure 1C), with the highest yield ratio (>20%) obtained when degumming was performed at 120 °C. By contrast, the yield ratio was little affected by the heating time (Figure 1C). The silk sericin proteins showed characteristic absorption bands at the mid-infrared regions of 1600–1700 cm^−1^ (amide I), 1480–1580 cm^−1^ (amide II), and 1230–1300 cm^−1^ (amide III) (Figure 1D) [1,5,12]. In particular, the amide I band, which corresponds to C=O stretching, was highly sensitive to the conformation type of the protein secondary structures [12]. Thus, careful peak assignment and evaluation of the abundance of the amide I sub-bands were quite useful for obtaining detailed information about protein secondary structures such as β-sheets, helices, β-turns, and random coils. A magnified spectral view of the amide I band showed broad vibrational absorptions centered at 1644 cm^−1^ for the examined sericin proteins.

Modification of silk sericin with 4HR resulted in a protein structure with increased absorption at ~1620 cm^−1^ (Figure 1E). The broad amide I band could be differentiated into several fine peaks by calculating the second derivative of the infrared absorption spectra (Figure 1F). These fine peaks could be assigned to β-sheet, random coil, helix, and β-turn structures [12]. Interestingly, the abundance of the β-sheet secondary structure at ~1620 cm^−1^ in sericin protein increased following pretreatment with 4HR, whereas the other secondary structures such as random coils, helices, and β-turns (at 1649, 1660, and 1682 cm^−1^, respectively) were notably reduced in abundance when compared with untreated sericin protein.

Examination of their UV spectra showed that 4HR and sericin had different peak patterns, with 4HR having an additional peak at 278 nm (Supplementary Figure S1). Thus, when compared with sericin alone, 4HR-bound sericin had an additional peak at 278 nm. This feature allowed release of sericin-bound 4HR to be analyzed spectrophotometrically. Incubation of sericin-bound 4HR in PBS resulted in rapid release of 4HR for 40 h, followed by a plateau (Supplementary Figure S2).

### 2.2. Silk Sericin Pretreated with 4HR Increases BMP-2 Expression in RAW264.7 Cells via a TLR-Mediated Signaling Pathway

**Figure 2:**
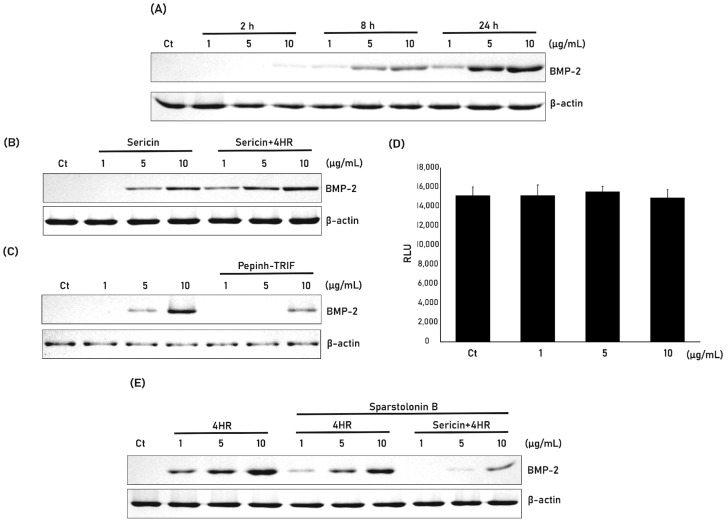
Effect of 4HR pretreatment of silk sericin on BMP-2 expression in RAW264.7 cells. (**A**) Western blot analysis of BMP-2 expression by RAW264.7 cells treated with 1, 5, and 10 µg/mL 4HR-sericin for 2, 8, and 24 h. (**B**) Western blot analysis of BMP-2 expression by cells treated with 1, 5, and 10 µg/mL sericin and 4HR-sericin. The relative expression level to β-actin was shown in Supplementary Figure S3. (**C**) Mechanisms by which sericin and 4HR induce BMP-2 expression. 4HR mainly induces BMP-2 expression by inhibiting histone deacetylase (HDAC), whereas sericin mainly induces BMP-2 via a Toll-like receptor (TLR) mediated pathway. TRIF is a mediator of the TLR signaling pathway. Pretreatment of cells with a peptide inhibiting TRIF (Pepinh-TRIF) prior to sericin + 4HR administration reduced BMP-2 expression, indicating that induction of BMP-2 expression by sericin + 4HR was mediated mainly by the TLR signaling pathway. (**D**) Effect of 4HR and sericin + 4HR on HDAC activity. Although 4HR is a strong inhibitor of HDAC activity, sericin + 4HR did not inhibit its activity (*p* > 0.05). (**E**) Effect of sparstolonin B, an inhibitor of TLR2 and TLR4, on BMP-2 expression induced by 4HR and sericin + 4HR. RAW264.7 cells were incubated with 1, 5, and 10 µg/mL 4HR, 4HR plus sparstolonin B or sericin + 4HR plus sparstolonin B, and BMP-2 expression was analyzed by western blotting.

Incubation of RAW264.7 cells with sericin pretreated with 4HR increased expression of BMP-2 in a dose-dependent manner (Figure 2A). The level of BMP-2 expression was slightly higher when RAW264.7 cells were treated with sericin + 4HR than with sericin alone (Figure 2B). The relative expression level of BMP-2 to β-actin was calculated and compared (Supplementary Figure S3). The difference between groups was statistically significant (*p* < 0.001). TRIF is a key signal transduction protein in the TRL pathway. Pretreatment of RAW264.7 cells with Pepinh-TRIF, a peptide that inhibits TRIF, reduced BMP-2 expression following treatment with sericin + 4HR (Figure 2C). 4HR is a strong HDAC inhibitor. If the free form of 4HR is released from sericin pretreated with 4HR, then treatment of RAW264.7 cells with sericin + 4HR would result in inhibition of HDAC activity. However, at the tested concentrations, HDAC activity did not differ significantly between cells treated with sericin + 4HR or sericin alone (Figure 2D).

Treatment of RAW264.7 cells with 4HR alone or sericin + 4HR increased expression of BMP-2 (Figure 2E). Pretreatment of these cells with sparstolonin B, an inhibitor of TRL-2 and TLR-4, prior to treatment with 4HR or sericin + 4HR, reduced expression of BMP-2, although the reduction was much greater in cells treated with sericin + 4HR than with 4HR alone.

### 2.3. Silk Sericin + 4HR Increases New Bone Formation in the Critically Sized Rat Calvarial Defect Model

**Figure 3:**
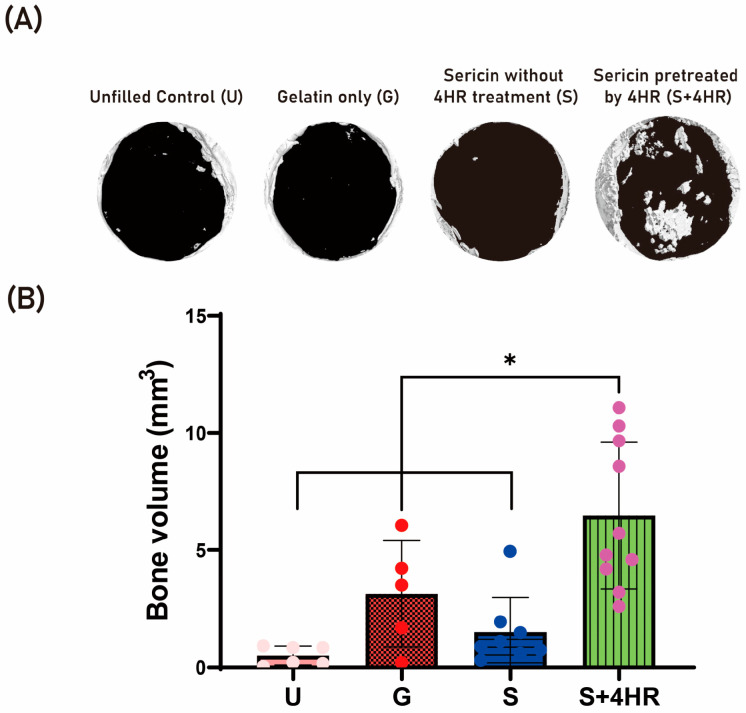
In vivo effects of sericin + 4HR and 4HR alone in the rat calvarian defect model. (**A**) Micro-CT results showing calcified areas in bone defects of rats receiving no implant (unfilled control [U] group), gelatin sponges alone (G group), or gelatin sponges containing sericin (S group) or sericin + 4HR (S+4HR group). (**B**) Quantitative analysis of bone volumes (BV) in the four groups. * *p* < 0.05.

The in vivo effects of sericin + 4HR and 4HR alone were evaluated in the rat calvarial defect model and the results compared with unfilled control (U) and gelatin only (G) groups from a previous publication [5]. Evaluation by micro-CT showed that the mean ± SD bone volumes (BVs) were 0.51 ± 0.40 mm^3^ in the U group and 3.13 ± 2.27 mm^3^ in the G group (Figure 3). In comparison, insertion of gelatin sponges containing silk sericin extracted at high temperature with (S+4HR group) and without (S group) 4HR pretreatment resulted in mean ± SD BVs of 6.47 ± 3.13 mm^3^ and 1.50 ± 1.48 mm^3^, respectively. The mean BV was significantly higher in the S+4HR group than in the S (*p* < 0.001), U (*p* < 0.001), and G (*p* = 0.028) groups.

**Figure 4:**
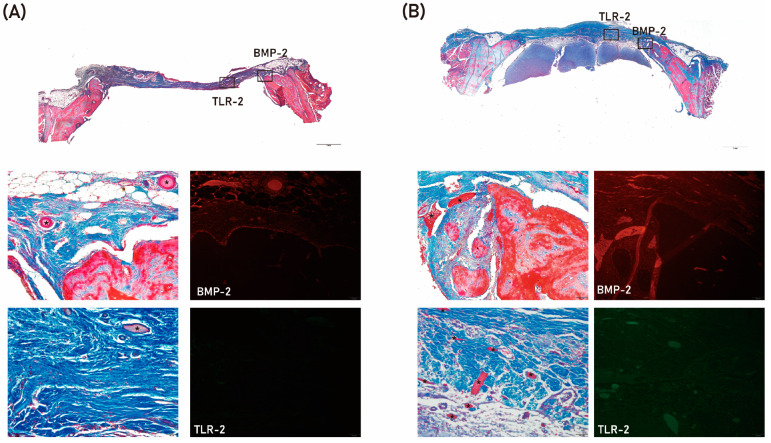
Histologic and immunofluorescence analysis of tissue samples from the S group (**A**) and the S+4HR group (**B**). Tissue sections were stained with Masson trichrome and viewed at magnifications of ×20 and ×200. BMP-2 and TLR-2 expression are shown as rectangles on the low magnification views. Remaining grafts (*) were stained red.

The results of histologic analysis are shown in Figure 4. Residual defects were smaller in the S+4HR group than in the S group. In addition, expression of BMP-2 and TLR-2 was higher in the S+4HR group than in the S group (Figure 4). BMP-2 and TRL-2 were mostly localized together adjacent to the area of new bone formation (Supplementary Figure S4). The expression level of runx2 was also higher in the S+4HR group than those in the S group (Supplementary Figure S5). On comparing the number of runx2 positive nuclei between groups, the positive nuclei in the S group were 21.17 ± 18.89 and those in the S+4HR group were 76.83 ± 29.25. The difference between groups was statistically significant (*p* = 0.003).

**Figure 5:**
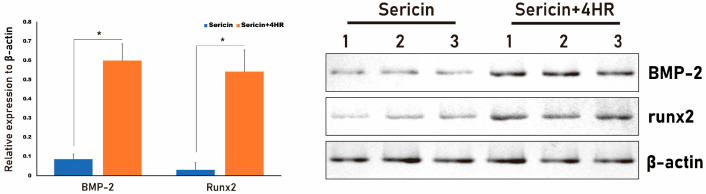
Western blot analysis of BMP-2 and runx2 proteins in tissue samples from the S and S+4 HR groups. Expression of BMP-2 and runx2 was significantly higher in the S+4HR group than in the S group. * *p* < 0.05.

Total proteins were extracted from tissue samples randomly selected from each group. The relative expression of BMP-2 (0.60 ± 0.09 vs. 0.09 ± 0.03, *p* = 0.001) and runx2 (0.54 ± 0.11 vs. 0.03 ± 0.04, *p* = 0.002) was significantly higher in the S+4HR group than in the S group (Figure 5).

## 3. Discussion

The β-sheet structure content negatively affects the solubility of sericin 11. However, the number of β-sheet structures was higher in water extracts of sericin pretreated with 4HR than in water extracts of untreated sericin control (Figure 1F). Compared with degumming at low temperature (37 °C to 70 °C), degumming at high temperature (100 °C to 120 °C) markedly increased the yield ratio of sericin (Figure 1C). Moreover, expression of BMP-2 was higher in RAW264.7 cells treated with sericin extracted by 4HR pretreatment at high temperature than with sericin extracted at low temperature with centrifugation (Figure 2B). Inhibition of the TRL signaling pathway by either pepinh-TRIF or sparstolonin B reduced the expression of sericin + 4HR-induced BMP-2 in RAW264.7 cells (Figure 2C,E). These findings suggest that sericin extracted after 4HR pretreatment induces BMP-2 expression in RAW264.7 cells via a TLR-mediated signaling pathway. Moreover, grafting gelatin sponges containing sericin extracted after 4HR pretreatment into bone defects significantly enhanced new bone formation compared with the grafting of gelatin sponges containing sericin extracted at high temperature without 4HR pretreatment (*p* < 0.05; Figure 3). Collectively, these findings indicate that 4HR pretreatment before high temperature degumming improves not only the yield ratio of sericin, but enhances BMP-2 expression compared with sericin extracted at low temperature and centrifugation.

**Figure 6:**
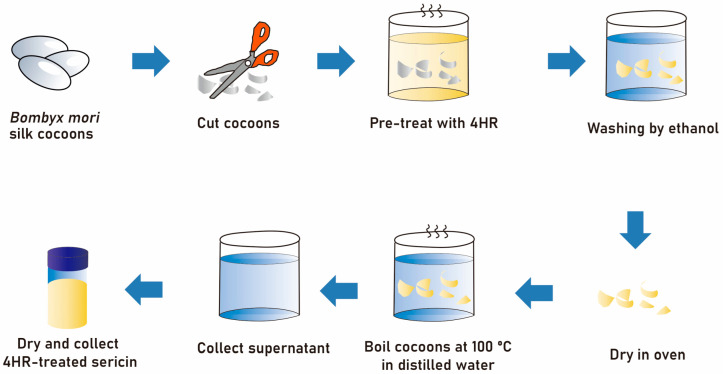
Schematic drawing of the method used for 4HR pretreatment of silk cocoons and subsequent extraction of silk sericin.

BMP-2 induction by 4HR-pretreated sericin was mediated mainly by the TLR signaling pathway [5], whereas 4HR-induced TGF-β family expression was mediated by HDAC inhibition [13]. Because hyperacetylated histone is required to induce the expression of genes belonging to the TGF-β family, HDAC inhibits their expression [14]. 4HR is an inhibitor of HDAC [14], thereby enhancing the acetylation histone [13]. Hyperacetylated histone can bind to gene promoters, thereby enhancing transcription of genes encoding TGF-β family proteins such as BMP-2 [13]. By contrast, the present study shows that inhibiting the TLR signaling pathway by administration of S+4HR suppressed the expression of BMP-2 (Figure 2C,E). The free form of 4HR should be removed by washing with 100% ethanol (Figure 6). 4HR bound to sericin likely does not bind to HDAC as it cannot enter the substrate binding site of the latter. However, 4HR-pretreated sericin contains more β-sheets (Figure 1F), suggesting that 4HR-pretreated sericin can bind to TLR and generate downstream signals. Pretreatment with sparstolonin B decreased BMP-2 expression induced by treatment with 4HR alone (Figure 2E) and this might be due to the effect of sparstolonin B on BMP-2 expression. Indeed, a previous study shows that sparstolonin B reduces BMP-2 expression in cancer cells [15]. There was no HDAC inhibition after S+4HR treatment (Figure 2D). Thus, 4HR-induced BMP-2 expression may be mainly activated by a TLR independent signaling pathway.

Chemical chaperones are commonly found in nature. The key functions of these chemical chaperones include (1) stabilization of structural proteins, (2) reduction of metabolic rates, and (3) suppression of mitochondrial respiration. These functions enable chemical chaperones to protect cells from environmental stresses [16]. Natural chemical chaperones produced by cells subjected to environmental stresses bind to many types of proteins, stabilizing their structure. 4HR is regarded as a chemical chaperone [7]. 4HR treatment of micro-organisms suppresses microbial proliferation and induces dormancy, with this dormant state characterized by low respiration and a thickened cellular membrane [17,18]. These micro-organisms are highly resistant to environmental stresses as their structural components are stabilized by 4HR [16]. Based on this property, 4HR has been used to preserve DNA structures [11]. P53 is an important transcription factor that induces cellular apoptosis; indeed, many cancers show mutations in the DNA binding domain of p53 [19]. The natural conformation of the DNA binding domain of p53 is a β-sheet structure [10]. 4HR treatment of cancer cells bearing mutant p53 results in the recovery of p53 transcriptional activity, potentially by restoring its β-sheet structure [10]. Similarly, the present study found that 4HR pretreatment increased the β-sheet structure in the sericin protein, with sericin-bound 4HR not released during the degumming process (Figure 1).

In this study, BMP-2 expression after sericin administration was examined in a monocytic line macrophage (Figure 2). Osteomacs are bone tissue-resident macrophages and have a pivotal role in wound healing [20]. Except for autogenous grafts, most grafts are foreign material and should interact with tissue-resident macrophages. A bone graft having osteoimmunomodulatory property can stimulate osteomacs and let these macrophages stimulate osteoblasts [21]. Bioactive sericin has this property [5]. In this study, 4HR-pretreated sericin could stimulate RAW264.7 cells to increase the expression level of BMP-2 (Figure 2A,B). In the animal model, the S+4HR group showed significantly higher BV than that in the S (*p* < 0.001), U (*p* < 0.001), and G (*p* = 0.028) groups (Figure 3). Runx2 is an active osteoblast marker [22]. The expression of BMP-2 can be observed in macrophages and osteoblasts [23]. The expression level of runx2 and BMP-2 was significantly higher in the S+4HR group compared to the S group (*p* < 0.05; Figure 5).

The present study has several limitations. First, some of the FT-IR peaks of silk sericin and 4HR overlapped (Supplementary Figure S1), which prevented determination of the exact amount of 4HR in 4HR-pretreated sericin. Although its peak at 278 nm was characteristic of 4HR, silk sericin also had a weak peak in this area (Supplementary Figure S1). Second, BV data from the unfilled control and gelatin-only groups were obtained from our previous study [5]. Although the rat strain and housing conditions were identical to those in the present study, the experimental conditions in the two studies may have differed slightly, potentially affecting between-group comparisons. In particular, BV in the previous study was measured by an outside laboratory, whereas BV in the present study was measured in-house; therefore, differences among observers may have affected the final results. Third, 4HR itself could have induced BMP-2 expression (Figure 2E). However, in the present study HDAC was only slightly inhibited by sericin + 4HR (Figure 2D). Therefore, the increased expression of BMP-2 is likely due primarily to 4HR-bound sericin. Additional studies, however, are required to confirm these findings.

## 4. Materials and Methods

### 4.1. Sericin Extraction and Analysis of the 4HR Incorporation Ratio

4HR (CAT#: 209465; Sigma-Aldrich, St. Louis, MO, USA) had poor solubility in water, but was soluble in ethanol. Ethanol (CAT#: A995-4; Thermo Fisher Scientific, Waltham, MA, USA) was used as a solvent for 4HR. Silkworm cocoons of Bombyx mori (*Baegokjam*) were chopped into small segments, which were then immersed in a 0.05% solution of 4HR (Solution A) and stirred to increase incorporation of 4HR into the cocoons. The volume of the remaining solution (Solution B) was measured, and free 4HR was removed by washing in 100% ethanol (Solution C). The silk fragments were dried to remove ethanol and then boiled in distilled water under pressure (0.15 MPa) using an autoclave (Daihan Scientific, Wonju, Korea). The supernatant was collected and dried (Figure 6). The amount of 4HR incorporated into the cocoons was calculated using the equation: Final 4HR in cocoon (g) = [(4HR in Solution A (g) − 4HR in Solution B (g)) − 4HR in Solution C (g)]. A calibration curve was constructed spectrophotometrically at 278 nm (Thermo Fisher Scientific).

### 4.2. Scanning Electron Microscopy (SEM)

Cocoons that had incorporated 4HR were fixed with carbon tape and coated with metal using an Ion Sputter Coater (G20, S3 Alliance Ltd., Londonderry, UK) under vacuum. The surfaces of the cocoons were evaluated using SEM (Emcrafts Ltd., Gwangju, Korea) and Virtuoso software (version 1.0.0).

### 4.3. Determination of Yield

Silk sericin was extracted at temperatures of 37, 70, 100, or 12 °C for 1, 3, or 5 h. The extracts were lyophilized and their dry weight measured. The extraction yield was determined using the equation: Extraction yield (%) = [Lyophilized extract (g)/cocoon weight (g)] × 100.

### 4.4. Release of 4HR-Incorporated Sericin from Solution

This method has been described in detail [24]. Briefly, samples (1 g of graft) were added to phosphate buffered saline (PBS) and stirred at 37 °C. Aliquots were collected at 0.5, 1, 2, 3, 6, 12, 24, 30, and 94 h. The concentrations of released 4HR were measured spectrophotometrically at 278 nm (Thermo Fisher Scientific).

### 4.5. FT-IR Spectroscopy

Solutions were analyzed using a Fourier transform spectrometer (Vertex 80, Bruker Optics, Billerica, MA, USA). Spectra were recorded in the range of 1000 to 1800 cm^−1^ at a spectral resolution of 4 cm^−1^ using a deuterated L-alanine-doped triglycine sulphate detector. Scans were repeated 128 times and the data for each spectrum were averaged.

### 4.6. Cell Cultures and Sericin Treatment

RAW264.7 murine macrophages were obtained from the Korean Cell Line Bank (No. 40071) and treated with two types of sericin. One type was extracted following 4HR pretreatment, as described above, whereas the other was extracted by the same method, but omitting 4HR-pretreatment. The cells were incubated for 2, 8, and 24 h with 1, 5, or 10 μg/mL of each type of sericin, or with the same volume of vehicle. The cells were collected, and BMP-2 expression was analyzed by western blotting using antibodies specific for BMP-2 (CAT# sc-137087) and β-actin (CAT#: sc-47778), both from Santa Cruz Biotechnology (Santa Cruz, CA, USA).

To analyze the signaling pathway responsible for the activity of sericin + 4HR, cells were treated with a peptide that inhibited Toll/interleukin-1 receptor-domain-containing adapter-inducing interferon-β (TRIF; CAT# tlrl-pitrif; InvivoGen, Hong Kong) or with a Pepinh-Control prior to treatment with 1, 5, or 10 μg/mL sericin + 4HR. The cells were collected and changes in BMP-2 expression were analyzed by western blotting. Relative expression level of BMP-2 to β-actin was calculated and compared. In addition, cells were treated with sparstolonin B (CAT# SML1767; Sigma-Aldrich), an inhibitor of TLR-2 and TLR-4 [25] prior to treatment with 1, 5, or 10 μg/mL 4HR or sericin + 4HR. 4HR is a histone deacetylase inhibitor [26]; thus, if 4HR was present in sericin as a free form (not bound to sericin) it could inhibit the activity of histone deacetylase. Enzyme activity was measured with a commercially available kit (CAT#: ab156064, Abcam, Cambridge, UK).

### 4.7. Animals and Experimental Design

The animal study was approved by the Gangneung-Wonju National University for Animal Research (GWNU-2022-7; approval 29 April 2022), with all procedures performed according to the guidelines for laboratory animal care of Gangneung-Wonju National University. Specific pathogen-free/viral antibody-free Sprague-Dawley rats, aged 8 weeks, were obtained from Orientbio Inc. (Sungnam, Korea) and allowed to acclimate for 1 week before experimentation.

Rats were anesthetized with a combination of 0.5 mL zoletil (125 mg/mL; Bayer Korea, Seoul, Korea) and 0.5 mL xylazine (Rompun; Bayer Korea). Their scalps were sterilized with povidone, and an incision made on the mid-sagittal area of the calvaria. Supra-periosteal dissection was performed to remove the periosteum during the defect preparation step. A critically sized defect was prepared using a trephine burr measuring 8.0 mm in diameter. After removing the calvarial bone with the periosteum, a graft comprising approximately 50 µg of sericin (Group S) or sericin + 4HR (Group S+4HR) loaded onto a gelatin sponge (Cutanplast Dental^®^, Uniplex, Sheffield, UK) was inserted into the defect. Wounds were closed with 3-0 black silk. Rats were administered an antibiotic (gentamicin; Daesung, Yiwang, Korea) and an analgesic (tolfenamic acid; Samyang Anipharm, Seoul, Korea) for 48 h postoperatively. During the first 24 h, two animals from Group S died; thus, Group S comprised eight rats and Group S+4HR comprised 10 rats. After 8 weeks, all rats were sacrificed, and specimens from their calvaria were processed for further analysis.

### 4.8. Micro-Computerized Tomography (mCT)

To avoid unnecessary animal sacrifice, data for six rats that underwent calvarial bone removal but were not otherwise treated (unfilled control Group U), and five rats that underwent insertion of a gelatin sponge following bone removal (Group G), were obtained from a previous study (GWNU-2018-14) [5]. The experimental conditions (weight, source, genetic background, age of these animals, housing and surgical procedure, etc.) of previous study (GWNU-2018-14) were identical to the current experiment (GWNU-2022-7).

Calvarial samples measuring 10 × 10 × 0.5 mm were placed in a sample holder and analyzed by μCT50 (Scanco Medical, Brüttisellen, Switzerland) in the Center for Research Facilities at Gangneung-Wonju National University. The position of each sample was confirmed. Raw images were analyzed to determine regions of interest (ROI) based on the shape and size of the original defect. The bone volume (BV) in each ROI was calculated by a CT Analyzer (version1.17.7.2+, Skyscan, Kartuizersweg, Belgium). The images were analyzed with lower and upper grayscale thresholds set to 48 and 255, respectively.

### 4.9. Histology, Immunohistochemistry, and Western Blotting of Tissue Samples

Following mCT image analysis, all specimens were incubated in decalcification solution for 5 days at room temperature. Each decalcified specimen was divided into halves, with one half embedded in paraffin and the other used for protein extraction and western blotting. The paraffin-embedded samples were cut at a thickness of 5 μm and subjected to trichrome staining with a commercially available kit (CAT# HT15, Sigma-Aldrich), and immunostaining with Alexa Fluor^®^-tagged antibodies specific to TLR-2 (CAT# sc-21759 AF488) and BMP-2 (CAT# sc-137087 AF680), both from SantaCruz Biotechnology (dilution ratio = 1:100). They were applied to slides and incubated in a humidified dark chamber overnight at 4 °C. After washing, each slide was mounted with fluorescence mounting medium (Dako, Glostrup, Denmark). The mounted slide was examined with both Stellaris 5 (Leica Microsystems, Wetzlar, Germany) at the Center for Scientific Instruments, Gangneung-Wonju National University and Olympus fluorescent microscope (BX51, Tokyo, Japan). The immunostaining for runx2 was done with the antibody for runx2 (CAT# sc-390351, SantaCruz Biotechnology; dilution ratio = 1:100). After application of primary antibody and incubation, ready-to-use secondary antibody (Dako^®^ Real Envision^®^, Dako) was applied and washed with PBS. Chromogen for runx2 was DAB and positive area was shown as a brown color. Runx2 positive nuclei were counted in both groups using image analysis program (Sigma Scan Pro 5.0, Chicago, IL, USA) and compared. Proteins extracted from tissue samples were subjected to western blotting with antibodies specific to runx2 (CAT# sc-390351) and BMP-2 (CAT# sc-137087), both from SantaCruz Biotechnology. Relative expression level of each protein to β-actin was calculated and compared.

### 4.10. Statistical Analysis

Results were reported as the mean ± standard deviation (SD). Differences among multiple groups were analyzed by analysis of variance, with subsequent differences between groups analyzed by post-hoc Bonferroni’s tests. All statistical analyses were performed using SPSS12.0K software for Windows (Chicago, IL, USA), and bar graphs with scatter plots were drawn using Prism 9 software for Windows (GraphPad Software, San Diego, CA, USA). The level of significance was set at *p* < 0.05.

## 5. Conclusions

The present study showed that 4HR pretreatment increased the β-sheet structure in degummed silk sericin. Compared with untreated sericin, 4HR-pretreated sericin increased BMP-2 expression by RAW264.7 cells. 4HR pretreatment improved the yield of biologically active sericin when compared to the extraction of sericin at room temperature. Finally, when compared to untreated sericin grafts, 4HR-pretreated sericin grafts increased new bone formation in the critical-sized rat calvarial defect model.

## Data Availability

Not applicable.

## Supplementary Materials

The supporting information can be downloaded at: https://www.mdpi.com/article/10.3390/ijms24010150/s1.
