# Supplementary material for: 4-Hexylresorcinol Treatment before Degumming Increases the β-Sheet Structure of Silk Sericin and BMP-2 Expression in RAW264.7 Cells
Source: Int J Mol Sci. 2022 Dec 21;24(1):150. doi: 10.3390/ijms24010150 (PMC9820107; doi:10.3390/ijms24010150)

4-Hexylresorcinol Treatment before Degumming Increases the β-Sheet Structure of Silk Sericin and BMP-2 Expression in RAW264.7 Cells

Ji Hae Lee [1], HaeYong Kweon [1], Ji-Hyeon Oh [2], Yei-Jin Kang [2], Dae-Won Kim [3], Won-Geun Yang [4], Weon-Sik Chae [4] and Seong-Gon Kim [2,*]

[1] Industrial Insect and Sericulture Division, National Institute of Agricultural Sciences, RDA, Wanju 55365, Republic of Korea
[2] Department of Oral and Maxillofacial Surgery, College of Dentistry, Gangneung-Wonju National University, Gangneung 28644, Republic of Korea
[3] Department of Oral Biochemistry, College of Dentistry, Gangneung-Wonju National University, Gangneung 28644, Republic of Korea
[4] Daegu Center, Korea Basic Science Institute, Daegu 41566, Republic of Korea
* Correspondence: kimsg@gwnu.ac.kr; Tel.: +82-31-640-2468

Supplementary Figure S1. Examination of UV spectra.

Examination of their UV spectra showed that 4HR and sericin had different peak patterns. (A) 4HR has an additional peak at 278 nm. (B) Sericin has only one peak at 230 nm. Accordingly, 4HR incorporated sericin would have 2 peaks at 230 and 278 nm. Using this feature, the release pattern of 4HR incorporated sericin could be anticipated.

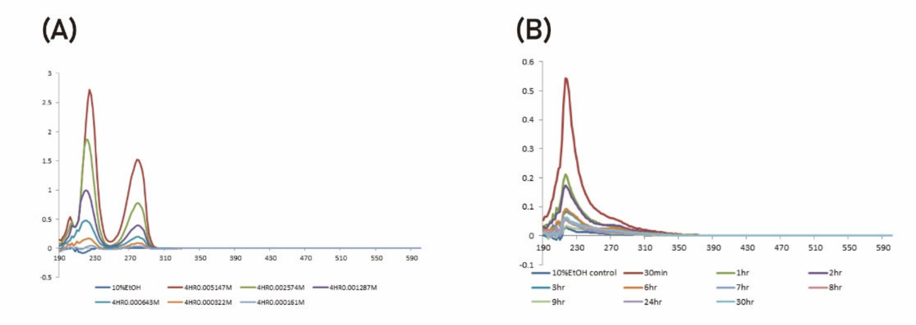

Supplementary Figure S2. Release of 4HR from 4HR-sericin in solution.

Sericin was incorporated into 4HR, with the resulting protein incubated in PBS for up to 94 h. The concentration of 4HR in PBS was determined spectrophotometrically at 278 nm. After 94 h 43% of the sericin-bound 4HR was released into the solution.

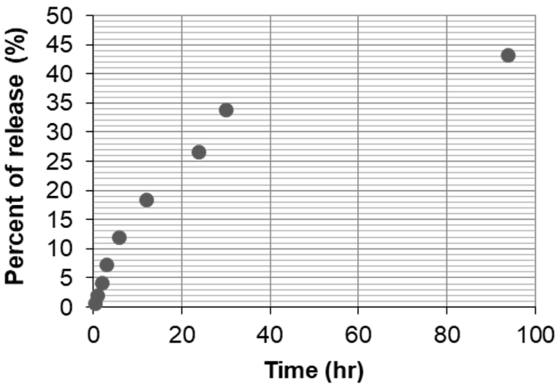

Supplementary Figure S3. Relative expression level of BMP-2 in Figure 2B.

The relative expression level of BMP-2 was compared between groups. The difference between groups was statistically significant (*P<0.001).

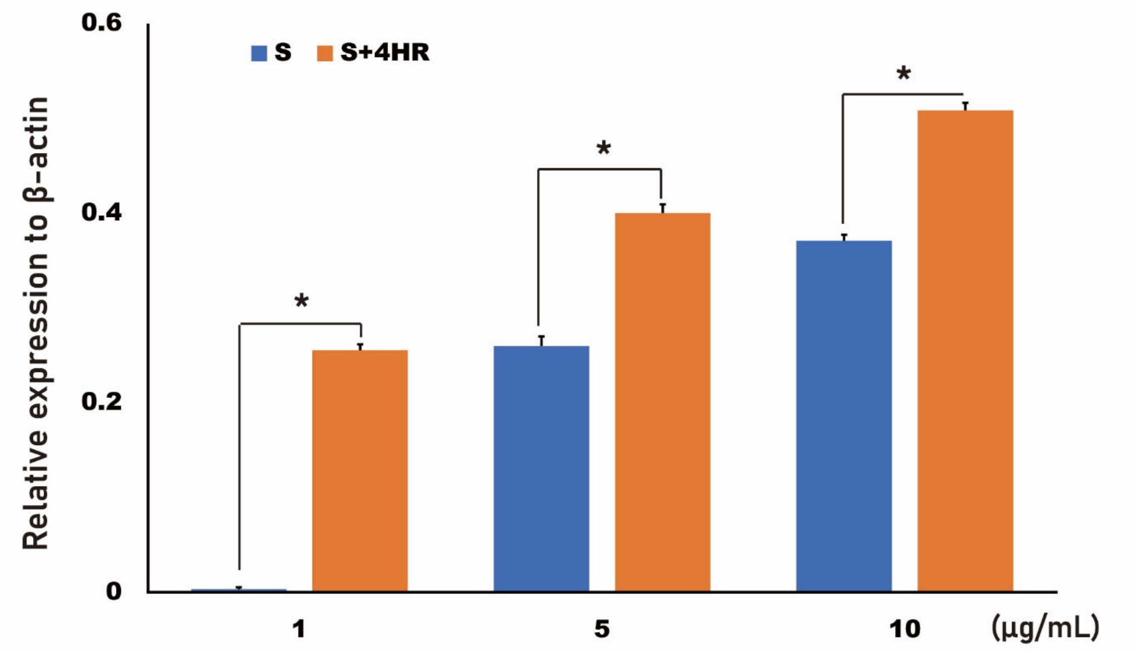

Supplementary Figure S4. Confocal microscopic images for BMP-2 and TLR-2.

(A) The expression level of BMP-2 and TLR-2 was low in sericin only group.

(B) The expression level of TLR-2 (green) was higher in sericin+4HR group. The expression of BMP-2 (red) was also observed. Some areas showed co-localization of TLR-2 and BMP-2.

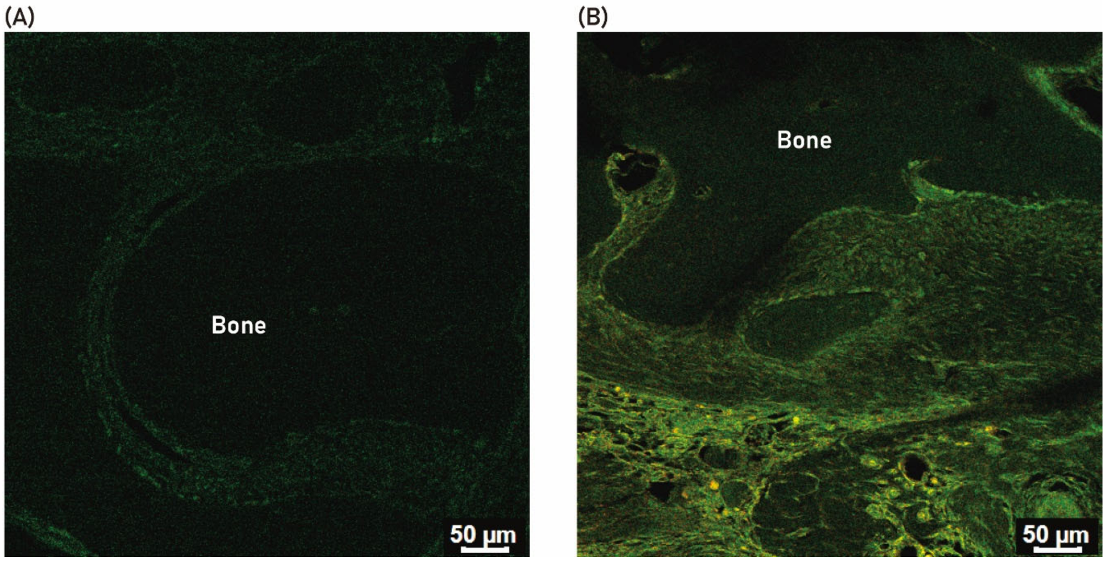

Supplementary Figure S5. The expression of runx2 in the tissue samples.

(A) The expression of runx2 in the sericin only group. The expression of runx2 was mainly found in the bone lining cells.

(B) The expression of runx2 in the sericin+4HR group. The expression level of runx2 was much higher compared to those in the sericin only group.

(C) The number of runx2 positive nuclei was counted and compared between groups. The area of bone ingrowth was examined and the size of examined area was 350 μm x 260 μm. The positive nuclei in the S group were 21.17 ± 18.89 and those in the S+4HR group were 76.83 ± 29.25. The difference between groups was statistically significant (P=0.003).

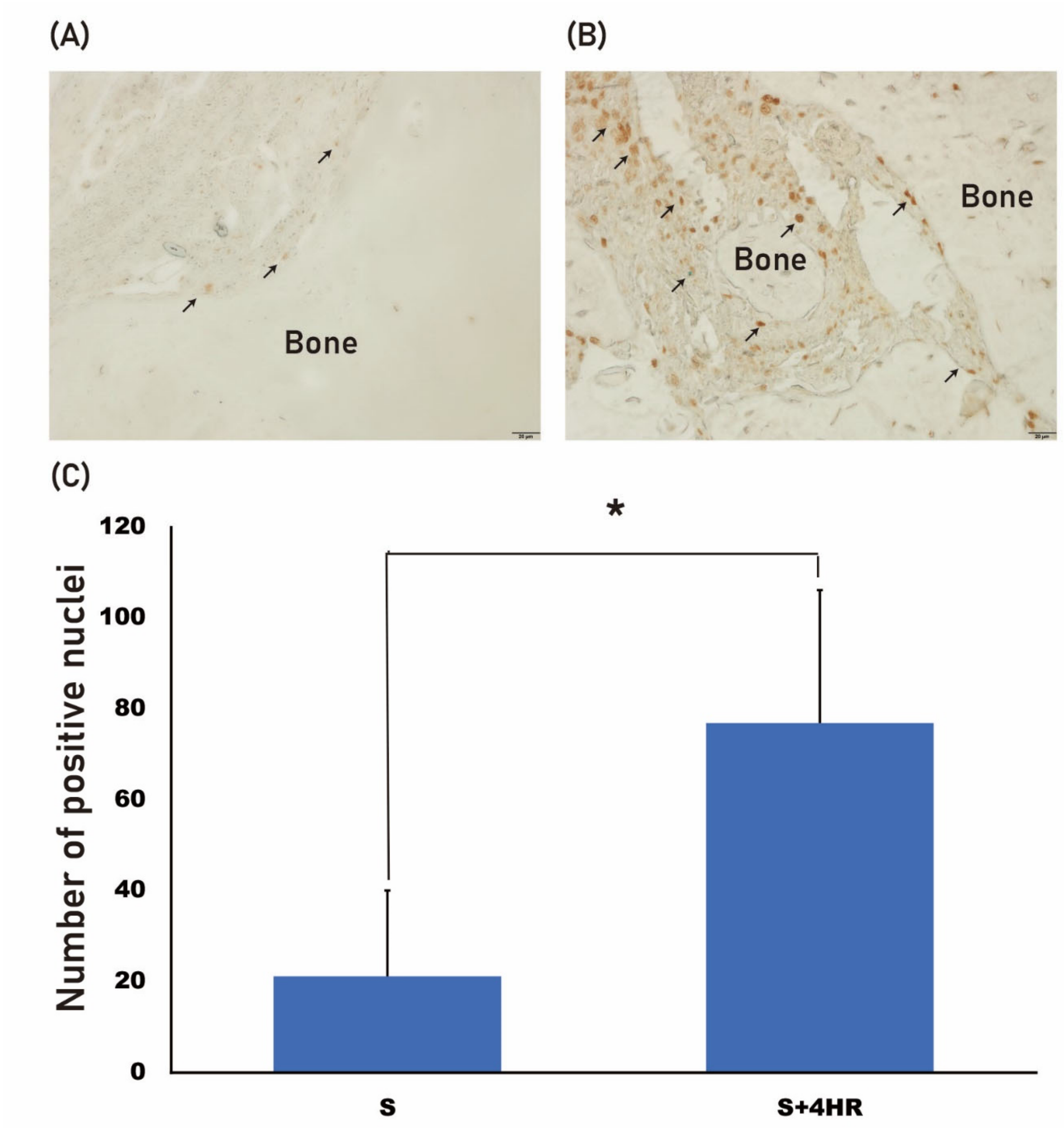

Supplement: Supplementary file 1 [file ijms-24-00150-s001.zip › ijms-2073371-supplementary.pdf]
